# Association between hepatic steatosis index and impaired fasting glucose: a multicenter retrospective cohort study in China

**DOI:** 10.3389/fendo.2025.1556169

**Published:** 2025-06-09

**Authors:** Yimei Chen, Jieying Han, Siwen Zhao, Yongjie Shi, Hongyun Jia, Songyao Lu, Juan Wu, Sicong Huang

**Affiliations:** ^1^ Department of Healthy Examination, The Second Affiliated Hospital, Guangzhou Medical University, Guangzhou, Guangdong, China; ^2^ Department of Clinical Laboratory, The Second Affiliated Hospital, Guangzhou Medical University, Guangzhou, Guangdong, China; ^3^ Department of Laboratory Medicine, Jieyang People’s Hospital, Jieyang, Guangdong, China

**Keywords:** impaired fasting glucose, hepatic steatosis index, retrospective cohort study, risk factors, Chinese adults

## Abstract

**Background:**

The Hepatic Steatosis Index (HSI) is a simple screening tool for adults with non-alcoholic fatty liver disease (NAFLD). While lipid and glucose metabolism are closely interrelated, few studies have examined the association between HSI and impaired fasting glucose (IFG). This study aims to investigate the relationship between HSI and IFG risk in a large Chinese cohort.

**Methods:**

This retrospective cohort study analyzed health examination data collected from 2010 to 2016 across 11 cities in China by the Rich Healthcare Group. Multivariable Cox regression and restricted cubic spline (RCS) analyses were used to evaluate the association between baseline HSI and IFG. Subgroup analyses were conducted to assess the robustness of the findings.

**Results:**

A total of 75,911 participants with a mean age of 40.9 ± 12.1 years were included, among whom 9,908 (13.1%) developed IFG. After adjusting for potential confounders, each one-unit increase in baseline HSI was associated with a 5% higher risk of IFG (HR=1.05, 95%CI). RCS analysis revealed that the increase of risk plateaued when HSI exceeded 35.31. Subgroup analyses demonstrated the stability of these findings.

**Conclusion:**

Elevated baseline HSI is a significant risk factor for IFG in Chinese adults. These findings highlight the potential utility of HSI in identifying individuals at risk of glucose dysregulation.

## 1 Introduction

Diabetes, a chronic metabolic disorder, has emerged as a significant global health challenge, with its prevalence increasing nearly fourfold over the past three decades. Approximately 463 million adults worldwide are affected, representing 9.3% of the population aged 20-79 (1). In China, the prevalence has risen dramatically from 0.67% in 1980 to 11.2% in recent years (2). Among diabetes cases, type 2 diabetes mellitus (T2DM) constitutes 90%, featuring high prevalence, low control rates, and numerous complications. Identifying high-risk populations and screening for risk factors are indispensable for early prevention.

Prediabetes, encompassing IFG and impaired glucose tolerance (IGT), represents an early phase of T2DM. Affecting 10.6% of the global population and 35.2% of Chinese adults (2), making it a critical target for prevention, as approximately 60% of diabetes cases progress from this stage within five years (3). Interventions such as lifestyle modifications and pharmacotherapy can reverse prediabetes and reduce the risk of developing T2DM (4). NAFLD is a liver manifestation of metabolic syndrome, affecting 25% of the global population. The prevalence rate in China has soared from 15% in 2003 to 32.1% in 2025 (5). NAFLD disrupts glucose metabolism through several key mechanisms: Hepatic insulin resistance develops primarily through lipid accumulation-induced impairment of IRS phosphorylation, while concurrent mitochondrial dysfunction activates the ROS-JNK pathway, further disrupting insulin signaling. These metabolic disturbances are compounded by adipokine imbalance, creating a pro-inflammatory milieu. Clinically, NAFLD patients show significantly higher prediabetes risk with 5% liver fat increase correlating to 8% β-cell function decline (HOMA-β). This bidirectional relationship is reinforced by hyperinsulinemia-driven activation of SREBP-1c mediated lipogenesis, thereby perpetuating a self-sustaining cycle of metabolic dysfunction (6).

Hepatic steatosis, closely associated with T2DM and metabolic syndrome, has garnered considerable attention. The HSI, a non-invasive tool integrating BMI and liver enzyme levels, effectively screens for NAFLD (7, 8). HSI has also been correlated with metabolic disorders, including T2DM (9), gestational diabetes (10), and diabetic complications (11). Nevertheless, its role in predicting prediabetes, particularly IFG, remains ambiguous.

This multicenter retrospective cohort study investigates the relationship between HSI and the risk of IFG in individuals with normal baseline fasting glucose levels. By addressing this knowledge gap, the study provides valuable insights into how variations in HSI influence the development of IFG and informs strategies for diabetes prevention.

## 2 Materials and methods

### 2.1 Data source

This study utilized raw data from a cohort of 211,833 individuals, originally collected by Chen et al. (12). The dataset includes comprehensive medical records of participants undergoing health evaluations. Data were obtained from the Dryad Digital Repository (DRYAD) database (https://datadryad.org/stash/dataset/doi:10.5061/dryad.ft8750v). Ethical approval for data collection was granted by the National Center for Health Statistics Institutional Review Board, and all procedures adhered to the Declaration of Helsinki. As the dataset is anonymized, informed consent was not required.

### 2.2 Study population

**Figure 1:**
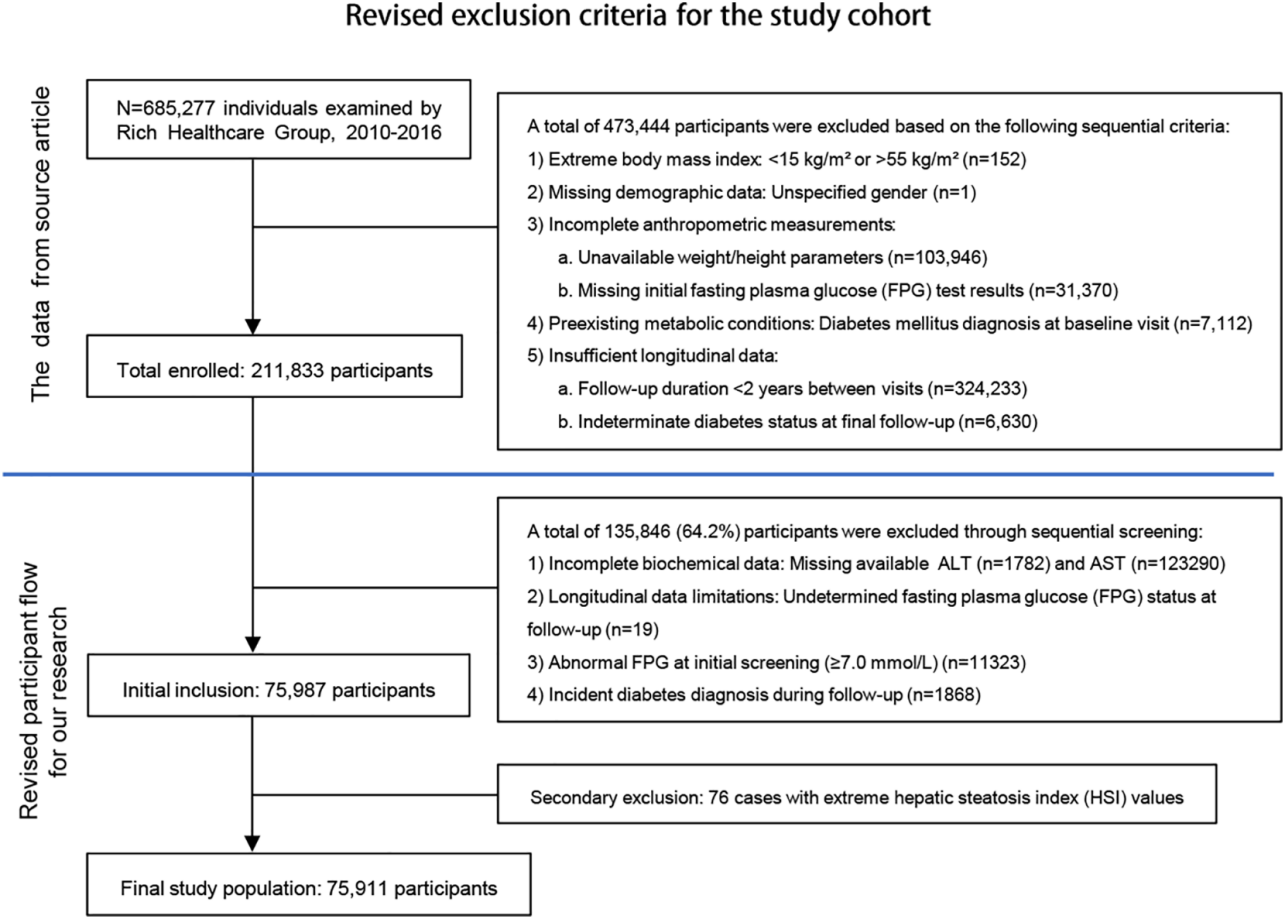
Flowchart of study population refinement.

This study analyzed data from 685,277 adults (≥20 years) without diabetes history in the Rich Healthcare Group’s 2010–2016 database (12), applying rigorous exclusion criteria to yield 75,911 participants (**Figure 1**). Missing continuous variables were handled via multiple imputation (5 iterations) after confirming missingness was completely at random (Little’s MCAR test *P* = 0.12), this approach remains vulnerable to violations if unmeasured confounders affected missingness. The complete-case analysis for categorical variables risks selection bias when missingness correlates with outcomes, and the limited imputation cycles may inadequately model complex biomedical relationships. Additionally, the 6-year observational period may not account for evolving diagnostic standards or measurement protocols. For the limitations of the above scheme, sensitivity analysis will be used subsequently to verify the robustness of the results. The data cleaning protocol and refine solution complied with Strengthening the Reporting of Observational Studies in Epidemiology (STROBE) guidelines (13).

### 2.3 Variables

Demographic data collected included age, sex, family history of diabetes, smoking status, and alcohol consumption. Physical examinations measured height, weight, and blood pressure, while laboratory tests assessed fasting plasma glucose (FPG), total cholesterol (TC), triglycerides (TG), high-density lipoprotein cholesterol (HDL-C), low-density lipoprotein cholesterol (LDL-C), blood urea nitrogen (BUN), creatinine clearance rate (CCR), aspartate aminotransferase (AST), and alanine aminotransferase (ALT). BMI was calculated as weight (kg) divided by height squared (m²). HSI was calculated as follows: *HSI = 8 × (ALT/AST ratio) + BMI (+2 for females)* (7).

### 2.4 Definitions

According to the American Diabetes Association (ADA) diagnostic criteria, IFG is characterized by FPG levels ranging from 5.6 mmol/L (inclusive) to below 7.0 mmol/L, in the absence of self-reported diabetes. This biochemical range specifically identifies individuals with prediabetic metabolic dysregulation (14). For study purposes, IFG onset time was operationally defined as the first detection date meeting diagnostic criteria during active surveillance (2010–2016), requiring at least two health examinations with >2-year intervals. Diabetes diagnosis required either FPG ≥ 7.0 mmol/L or self-reported diabetes history, creating mutually exclusive diagnostic categories that facilitated clear metabolic status classification.

### 2.5 Statistical analysis

Continuous variables were described as mean ± standard deviation for normally distributed data or median (interquartile range) for non-normally distributed data, while categorical variables were expressed as frequencies (percentages). Group differences were assessed using ANOVA for normally distributed continuous variables, Kruskal-Wallis tests for non-normally distributed variables, and chi-square tests for categorical variables.

Cox regression was selected to model time-to-event data for IFG risk factors, given its capacity to handle censored observations and provide hazard ratio estimates (15). Three adjustment models were implemented: 1) crude; 2) age/sex-adjusted; 3) fully-adjusted for clinical/laboratory/behavioral confounders (SBP, DBP, lipids, renal function, smoking/alcohol history, and diabetes family history). RCS with four knots (5th, 35th, 65th, 95th percentiles) were applied to evaluate potential non-linear HSI-IFG relationships, complemented by two-segment logistic regression with bootstrap-derived inflection points.

Subgroup analyses were conducted to explore effect modification across different groups, including age (<65 or ≥65 years) (16), sex, BMI (<24, 24-28, or ≥28 kg/m²) (17), family history of diabetes, alcohol consumption (current, former, or never), and smoking status (current, former, or never). Statistical analyses were performed using R 4.2.2 (http://www.R-project.org) and Free Statistics Analysis Platform 1.9.2 (http://www.clinicalscientists.cn/freestatistics), with two-sided *P*-values <0.05 considered statistically significant. As this study was based on existing data, sample size calculations were not performed.

## 3 Result

### 3.1 Baseline characteristics

This study included 75,911 participants with an average age of 40.9 ± 12.1 years. Among them, 33,843 (44.6%) were female, and 42,068 (55.4%) were male. Participants were divided into four quartiles based on HSI: Quartile 1 (Q1: 18.87–27.63), Quartile 2 (Q2: 27.64–30.53), Quartile 3 (Q3: 30.54–34.19), and Quartile 4 (Q4: 34.20–52.60). Compared to the other three groups, Quartile 4 exhibited significantly higher BMI, SBP, DBP, FPG, TC, TG, LDL-C, AST, ALT, BUN, and CCR levels. Additionally, the proportions of males, smokers, and alcohol consumers were higher in Quartile 4. Age, HDL-C levels, and family history of diabetes also differed significantly among the quartiles (*P* < 0.001) (**Table 1**).

**Table 1:** The baseline characteristics of participants.

Characteristics	Total	Q1 (18.87-27.63)	Q2 (27.64-30.53)	Q3 (30.54-34. 19)	Q4 (34.20-52.60)	*P-value*
Population	75911	18976	18979	18978	18978	
Age (year)	40.9 ± 12.1	37.7 ± 11.8	41.2 ± 12.3	43.2 ± 12.4	41.5 ± 11.3	< 0.001
Gender						< 0.001
male	42068 (55.4)	8031 (42.3)	8640 (45.5)	11083 (58.4)	14314 (75.4)	
female	33843 (44.6)	10945 (57.7)	10339 (54.5)	7895 (41.6)	4664 (24.6)	
BMI (km/m2)	23.0 ± 3.2	19.6 ± 1.5	21.9 ± 1.5	23.9 ± 1.7	26.7 ± 2.6	< 0.001
SBP (mmHg)	118.0 ± 15.9	112.5 ± 14.6	115.5 ± 15.2	119.5 ± 15.6	124.6 ± 15.5	< 0.001
DBP (mmHg)	73.5 ± 10.6	70.1 ± 9.5	71.6 ± 9.9	74.3 ± 10.5	77.9 ± 10.9	< 0.001
FPG (mmol/L)	4.8 ± 0.5	4.7 ± 0.5	4.8 ± 0.5	4.8 ± 0.5	4.9 ± 0.5	< 0.001
TC (mmol/L)	4.7 ± 0.9	4.4 ± 0.8	4.6 ± 0.9	4.7 ± 0.9	4.9 ± 0.9	< 0.001
TG (mmol/L)	1.0 (0.7, 1.5)	0.8 (0.6, 1.0)	0.9 (0.7, 1.3)	1.1 (0.8, 1.6)	1.5 (1.0, 2.2)	< 0.001
HDL-C (mmol/L)	1.4 ± 0.3	1.5 ± 0.3	1.4 ± 0.3	1.4 ± 0.3	1.3 ± 0.3	< 0.001
LDL-C (mmol/L)	2.7 ± 0.7	2.6 ± 0.6	2.7 ± 0.6	2.8 ± 0.7	2.9 ± 0.7	< 0.001
ALT (U/L)	23.3 ± 22.0	12.9 ± 6.9	16.5 ± 8.7	22.2 ± 13.0	41.7 ± 33.9	< 0.001
AST (U/L)	23.7 ± 12.4	21.1 ± 12.1	21.5 ± 7.8	23.3 ± 9.6	28.8 ± 16.6	< 0.001
BUN (mmol/L)	4.6 ± 1.2	4.5 ± 1.2	4.6 ± 1.2	4.7 ± 1.2	4.8 ± 1.2	< 0.001
CCR (umol/L)	70.9 ± 15.9	67.5 ± 14.7	68.7 ± 15.3	71.9 ± 16.8	75.3 ± 15.7	< 0.001
Family histroy of diabetes						< 0.001
No	74434 (98. 1)	18678 (98.4)	18601 (98)	18577 (97.9)	18578 (97.9)	
Yes	1477 ( 1.9)	298 (1.6)	378 (2)	401 (2. 1)	400 (2. 1)	
Smoking status						< 0.001
Current smoker	3349 ( 4.4)	584 (3. 1)	611 (3.2)	883 (4.7)	1271 (6.7)	
Ever smoker	817 ( 1. 1)	128 (0.7)	154 (0.8)	223 (1.2)	312 (1.6)	
Never smoker	14262 (18.8)	3848 (20.3)	3644 (19.2)	3500 (18.4)	3270 (17.2)	
Not recorded	57483 (75.7)	14416 (76)	14570 (76.8)	14372 (75.7)	14125 (74.4)	
Drinking status						< 0.001
Current drinker	413 (0.5)	89 (0.5)	77 (0.4)	108 (0.6)	139 (0.7)	
Ever drinker	3152 (4.2)	519 (2.7)	637 (3.4)	902 (4.8)	1094 (5.8)	
Never drinker	14863 (19.6)	3952 (20.8)	3695 (19.5)	3596 (18.9)	3620 (19. 1)	
Not recorded	57483 (75.7)	14416 (76)	14570 (76.8)	14372 (75.7)	14125 (74.4)	

### 3.2 Tendency of IFG Across HSI quartiles

**Figure 2:**
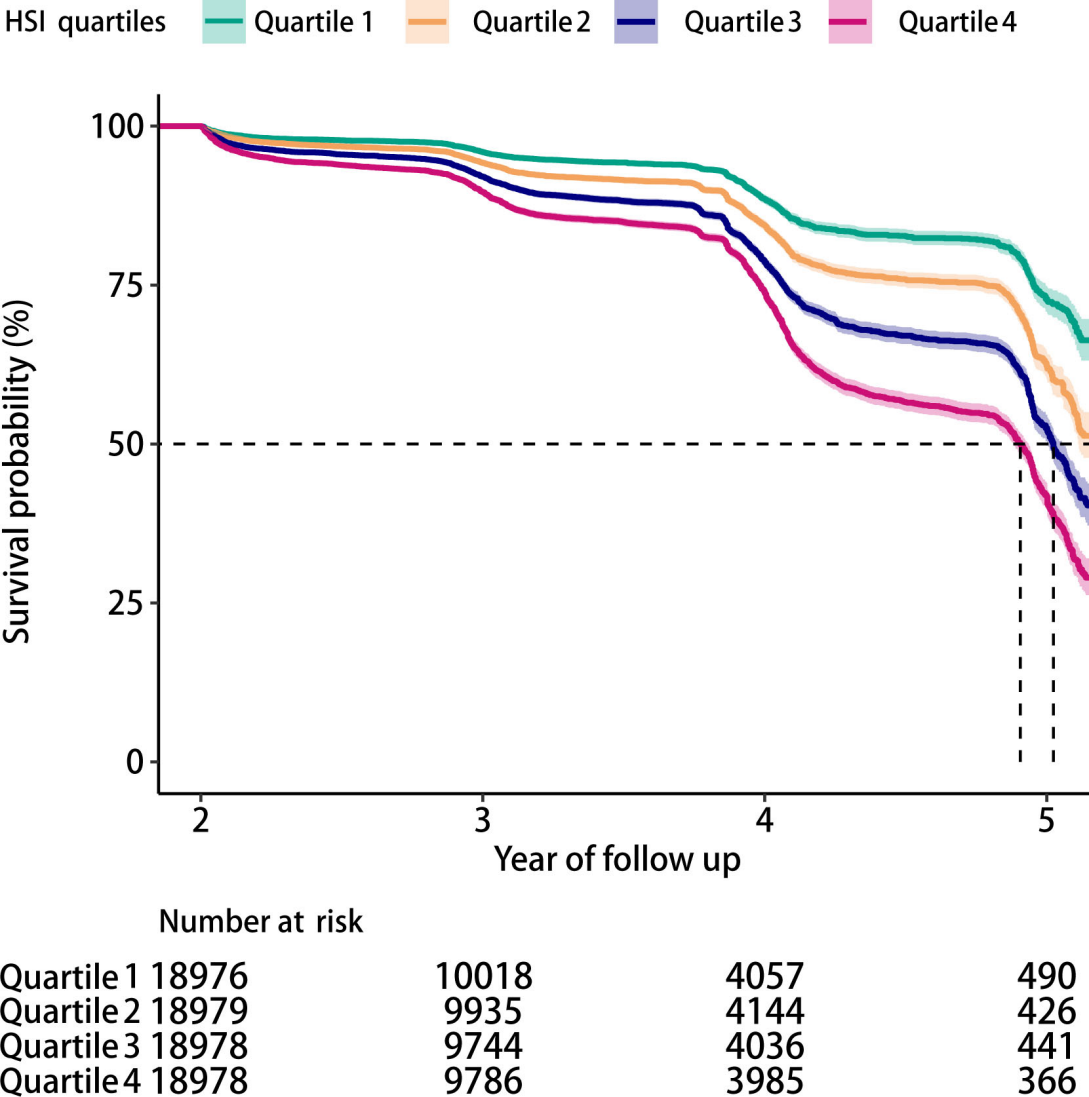
Kaplan-Meier survival curves showing the incidence of IFG across HSI quartiles (log-rank test, *P* < 0.05).

Over a mean follow-up period of 3.02 years, 9,908 participants (13.1%) developed into IFG. The Kaplan-Meier analysis disclosed significant differences in the risk of IFG across the HSI quartiles (*P* < 0.05), with a decreasing probability of maintaining normal glucose levels as the HSI increased (**Figure 2**).

### 3.3 Risk factors for IFG

Our univariate Cox regression analysis revealed significant associations between multiple baseline variables and incident IFG risk (all P < 0.001). Key metabolic parameters including age, blood pressure (SBP/DBP), lipid profiles (TC/TG/LDL-C), renal function markers (BUN/CCR), liver enzymes (AST/ALT), and anthropometric measures (BMI/FPG) demonstrated positive correlations with IFG development. Notably, male sex and modifiable lifestyle factors (smoking/alcohol consumption) also emerged as independent risk determinants. These results collectively illustrate the complex interplay between physiological markers and behavioral patterns in fasting glucose dysregulation, as comprehensively detailed in **Table 2**. The multifactorial pathogenesis of IFG underscores the necessity for integrated prevention strategies targeting both metabolic abnormalities and health behaviors.

**Table 2:** Results of univariate cox regression analysis.

Characteristics	HR (95%CI)	*P-value*
Age (year)	1.04 (1.04, 1.04)	< 0.001
Gender		< 0.001
male	Ref	
female	0.66 (0.63,0.69)	
SBP (mmHg)	1.03 (1.03, 1.03)	< 0.001
DBP (mmHg)	1.03 (1.03, 1.03)	< 0.001
FPG (mmol/L)	5.3 (5.04,5.57)	< 0.001
TC (mmol/L)	1.22 (1.2, 1.25)	< 0.001
TG (mmol/L)	1. 19 (1.18, 1.21)	< 0.001
HDL-C (mmol/L)	1.00(0.92, 1.08)	0.92
LDL-C (mmol/L)	1.23 (1.19, 1.27)	< 0.001
BUN (mmol/L)	1.13 (1.11, 1. 15)	< 0.001
CCR (umol/L)	1.0055 (1.005, 1.0059)	< 0.001
Family histroy of diabetes		0.358
No	Ref	
Yes	0.94 (0.82, 1.08)	
BMI (kg/m²)	1. 12 (1.12, 1. 13)	< 0.001
ALT (U/L)	1.004 (1.003, 1.004)	< 0.001
AST (U/L)	1.01 (1.00, 1.01)	< 0.001
Smoking status		< 0.001
Current smoker	Ref	
Ever smoker	0.9 (0.74, 1. 1)	
Never smoker	0.74 (0.67,0.81)	
Not recorded	0.88 (0.8,0.96)	
Drinking status		< 0.001
Current drinker	Ref	
Ever drinker	0.57 (0.44,0.73)	
Never drinker	0.52 (0.41,0.65)	
Not recorded	0.59 (0.47,0.74)	
HSI	1.07 (1.06, 1.07)	< 0.001

Dependent Variable: IFG. Independent Variables: Baseline characteristics (age, blood pressure, lipids, liver function markers, lifestyle factors). Significant Results: Positive associations were observed for all listed variables (*P* < 0.001).

### 3.4 Association between HSI and IFG

Given the intermittent follow-up design (median duration=3.02 years) precluding precise determination of IFG onset timing, we employed multivariable Cox regression to evaluate HSI-IFG risk associations (**Table 3**). The analysis revealed dose-dependent relationships: each unit increase in hepatic steatosis index (HSI) conferred a 5% elevated IFG risk (HR=1.05, 95%CI=1.05-1.06, P<0.001) in fully adjusted models, with quartile-based categorical analysis demonstrating consistent positive gradients. In pursuit of the stability of the Cox regression conclusion, complementary logistic regression assessing HSI-IFG prevalence associations was conducted. Crucially, both methodologies yielded concordant results: 1) equivalent magnitude of effect (OR=1.05 vs HR=1.05 per HSI unit); 2) identical statistical significance thresholds (P<0.001); 3) preserved quartile-response patterns (**Supplementary Table S1**). This rigorous analytical verification through complementary survival and cross-sectional frameworks substantiates the validity of our core findings regarding HSI’s predictive utility for IFG risk.

**Table 3:** Hazard ratios (HRs) for the association between HSI and IFG in different models.

Variable	Total	Crude Model		Model I		Modle II	
		HR (95%CI)	*P-value*	HR (95%CI)	*P-value*	HR (95%CI)	*P-value*
HSI	75911	1.07 (1.06, 1.07)	<0.001	1.06 (1.06, 1.07)	<0.001	1.05 (1.05, 1.06)	<0.001
HSI (Quartiles)							
Quartile 1	18976	Ref		Ref		Ref	
Quartile 2	18979	1.46 (1.36, 1.56)	<0.001	1.28 (1.2, 1.37)	<0.001	1.27 (1. 18, 1.36)	<0.001
Quartile 3	18978	2.067 (1.93, 2. 19)	<0.001	1.64 (1.54, 1.75)	<0.001	1.56 (1.46, 1.67)	<0.001
Quartile 4	18978	2.75 (2.59, 2.93)	<0.001	2.24 (2. 11, 2.39)	<0.001	1.97 (1.84, 2. 11)	<0.001
*P* for trend			<0.001		<0.001		<0.001

Crude Model: No covariates adjusted.

Model I:Adjusted for age and sex.

Model II: Adjusted for age, sex,SBP, DBP, TC, TG, HDL-C, LDL-C, BUN, CCR, smoking status, and family history of diabetes.

### 3.5 The nonlinear relationship between HSI and IFG

**Figure 3:**
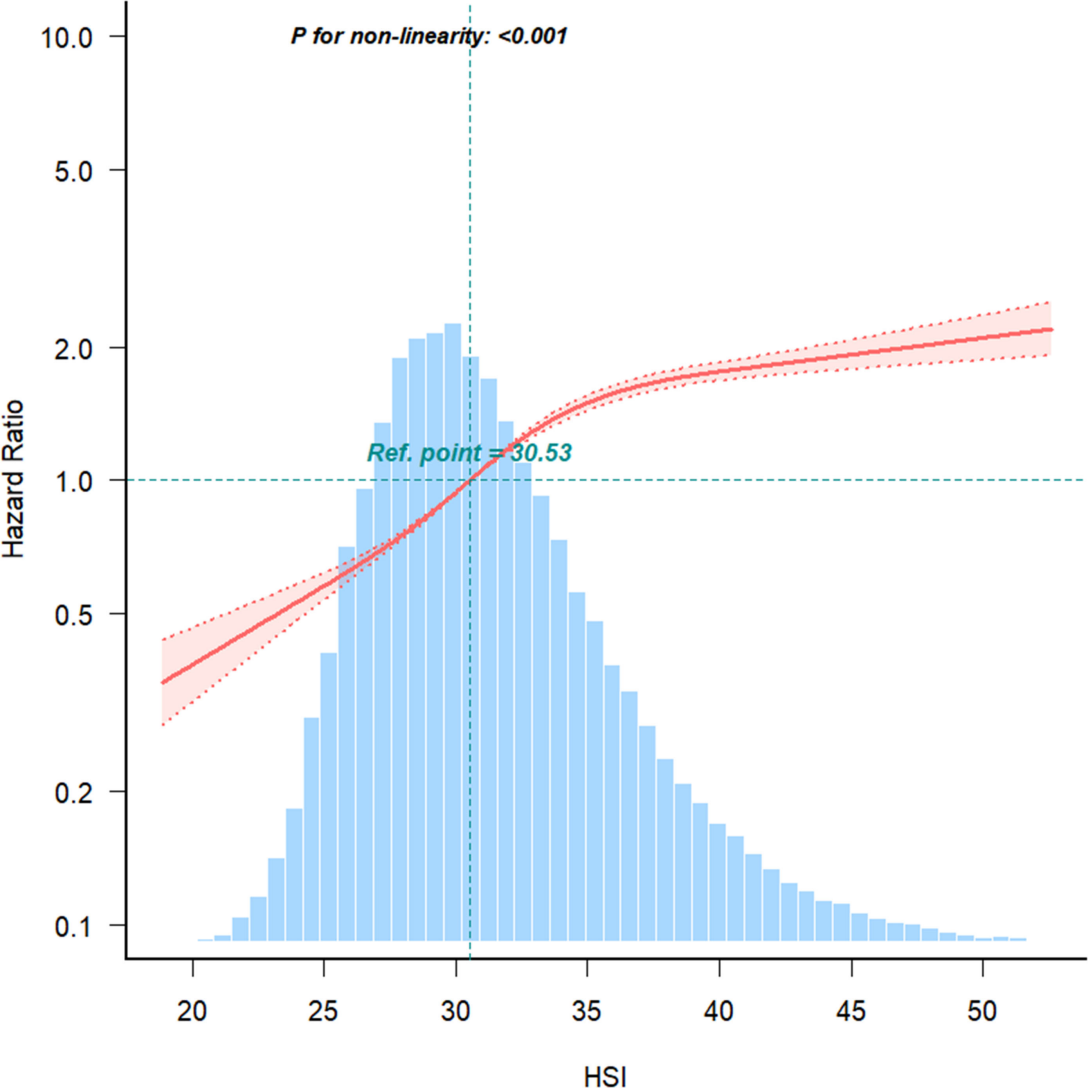
The nonlinear relationship between the relationship between HSI and IFG risk, with density plots of HSI distribution.

RCS analysis further demonstrated a non-linear relationship between HSI and IFG risk (*P* < 0.001) (**Figure 3**). Below an HSI threshold of 35.31, each 1-unit increase in HSI was associated with an 11% higher risk of IFG (HR = 1.11; 95% CI: 1.10–1.11). Above this threshold, the risk increased plateaued (**Table 4**).

**Table 4:** The result of the two-piecewise Cox regression model.

Variable	HR (95%CI)	*P-value*
Inflection points of HSI	35.31 (34.85, 35.77)	
< 35.31	1.11 (1.10, 1.11)	<0.001
> 35.31	1.02 (1.01,1.04)	<0.001
*P* for log likelihood ratio test	–	<0.001

### 3.6 Subgroup analysis

**Figure 4:**
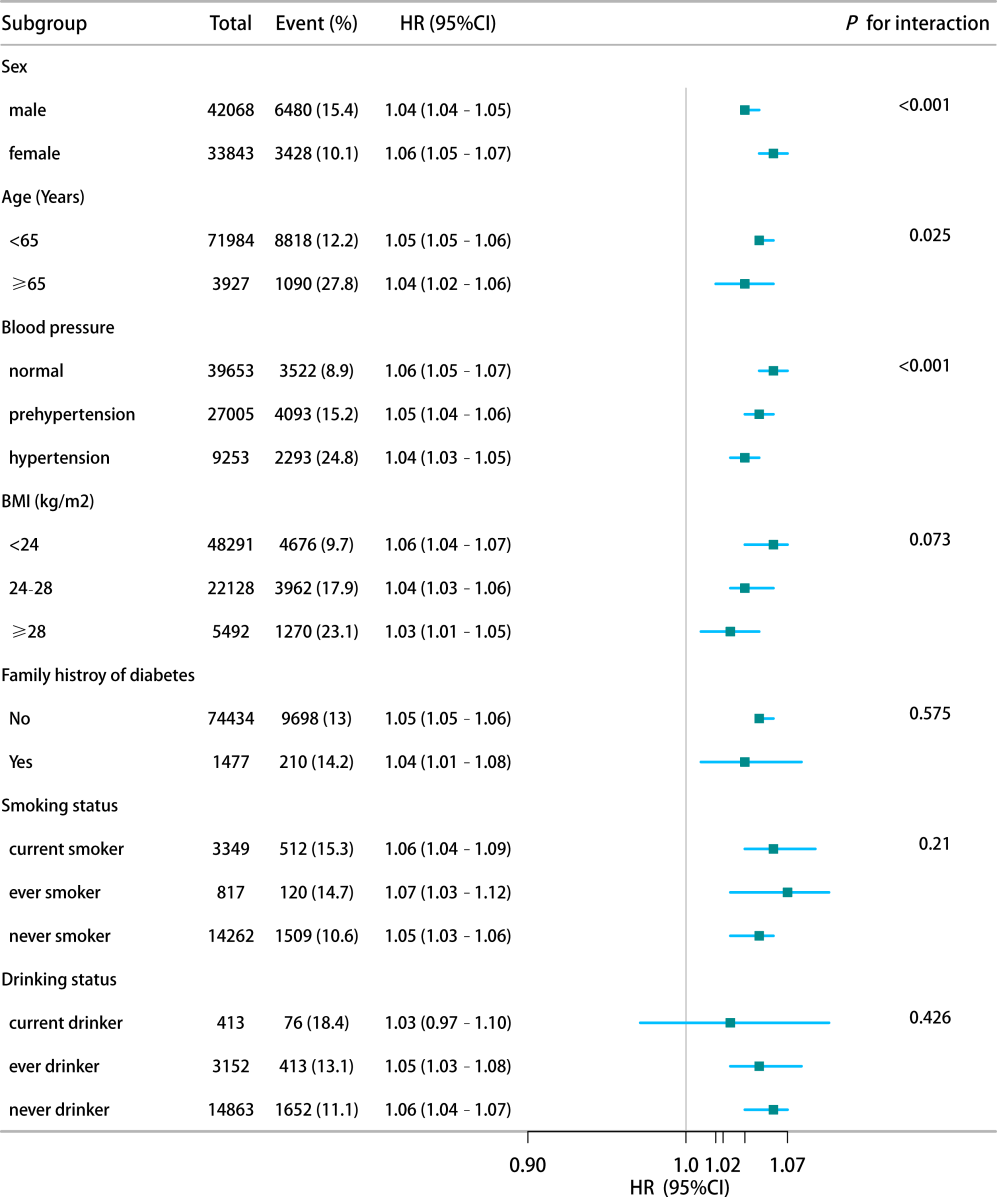
Stratified analysis of HSI-IFG risk associations by demographic and clinical characteristics.

Subgroup analyses were conducted to verify the robustness of the findings across different strata, including sex, age, blood pressure, BMI, family history of diabetes, smoking status, and alcohol consumption. In all subgroups, HSI remained positively associated with IFG risk. The association was stronger among female participants, aged <65 years and with normal blood pressure (**Figure 4**). These results consistently indicate that HSI is an independent risk factor for IFG.

## 4 Discussion

This large-scale retrospective cohort study employing biennial follow-up design (median 3.02 years) systematically examined the association between HSI and IFG. Our analysis revealed a novel nonlinear dose-response relationship with an inflection point at HSI=35.31: each unit increase below this threshold elevated IFG risk by 11%, while risk escalation attenuated to 5% per unit above this critical value. Notably, this association demonstrated significant interaction in women aged < 65 years. Methodologically, we implemented Cox proportional hazards models to accommodate irregular follow-up intervals, defining event time as first IFG detection and censoring date as last follow-up for non-converters (18). This approach outperformed logistic regression by effectively integrating heterogeneous follow-up durations and addressing right-censoring (19). Kaplan-Meier analysis corroborated the inverse relationship between HSI quartiles and normoglycemia maintenance probability (log-rank P<0.001), showing remarkable consistency with Cox model stratification. Standardized biochemical protocols coupled with sensitivity analyses ensured robustness against metabolic parameter measurement variability.

Prediabetes, characterized by IFG and/or IGT, carries a high risk of progression to T2DM. Global data indicate that 16% of IFG cases and 21% of IGT cases progress to T2DM within five years (20), and over 70% within a decade (21). The IFG incidence of 13.1% observed in our study exceeds prior reports, possibly due to differences in inclusion criteria and overlapping cases of simple or mixed IFG, as OGTT 2-hour glucose levels (<7.8 mmol/L) were not separately analyzed. Mechanistically, IFG primarily results from hepatic insulin resistance and β-cell dysfunction, whereas IGT is driven by peripheral insulin resistance, particularly in skeletal muscle (22), explaining the stronger correlation between HSI and IFG compared to IGT.

The relationship between HSI and prediabetes is non-linear, with the risk increase slowing when HSI exceeds 35.31. This phenomenon may be attributed to compensatory mechanisms, a biological saturation point, or the staged nature of clinical and pathological changes associated with hepatic fat accumulation (23). HSI has been closely linked to hepatic insulin resistance (24), which itself is influenced by various factors, including genetics (25), lifestyle (26), and metabolic conditions (27). When HSI surpasses 35.31, the slower risk increase for IFG may reflect the activation of compensatory mechanisms. Studies in high-fat diet-induced insulin resistance models in canines have demonstrated that compensatory hyperinsulinemia can occur independently of elevated blood glucose (28). Thus, beyond a certain threshold of HSI, more severe insulin resistance might be required to further elevate IFG risk. Pathologically, excessive hepatic fat accumulation disrupts insulin signaling, contributing to insulin resistance and increased glucose production (29). At HSI levels above 35.31, the liver may reach a biological saturation point, reducing its sensitivity to additional fat accumulation and insulin resistance.

The stronger association between HSI and IFG observed in individuals younger than 65 years may be due to the presence of multiple glucose-regulating factors in older adults, such as chronic inflammation (30) and reduced muscle mass (31), which diminish the relative contribution of HSI to IFG. Additionally, age-related factors, such as altered fat functionality and redistribution, complicate the impact of hepatic fat on glucose regulation (32). In younger individuals, where hepatic metabolic function and sensitivity to glucose-lipid regulation are relatively higher, hepatic fat accumulation likely exerts a more direct influence on fasting glucose regulation, resulting in a stronger correlation.

Among women, the association between HSI and IFG is more pronounced, possibly due to the regulatory role of estrogen on glucose metabolism. Hepatic fat accumulation may inhibit estrogen receptors (33), diminishing estrogen’s protective effects on insulin sensitivity (34). This interaction could accelerate the development of IFG in women, making changes in hepatic fat more significantly linked to fasting glucose dysregulation.

We further identified key demographic and metabolic factors associated with IFG risk, including male sex, obesity, hypertension, dyslipidemia, elevated transaminase levels, smoking, and alcohol consumption. These findings align with prior research highlighting obesity and chronic low-grade inflammation as drivers of insulin resistance and glucose dysregulation (35, 36). Obesity-related changes, such as altered gut microbiota and adipokine imbalance, exacerbate insulin resistance, leading to prediabetes. Effective interventions, including diet, aerobic exercise, and resistance training, have been shown to reverse prediabetes and normalize glucose levels (37, 38).

Liver function emerges as a pivotal factor in glucose regulation, with studies consistently linking elevated ALT, AST, and GGT levels to prediabetes and T2DM risk (39). Liver fat accumulation, a key feature of hepatic steatosis, impairs hepatic insulin sensitivity and triggers systemic inflammation, contributing to glucose dysregulation (40, 41). In our study, HSI, which incorporates ALT, AST, and BMI, was a strong predictor of IFG risk, particularly in younger individuals. This suggests that HSI reflects early hepatic metabolic disturbances that precede systemic glucose abnormalities.

While Wu et al. recently evaluated HSI’s predictive value for glycemic transitions (regression to normoglycemia vs diabetes progression) in high-risk populations with baseline IFG (42), our study extends this investigation to initially normoglycemic individuals. We demonstrate that elevated HSI predicts IFG development (5% increased risk per unit, HR=1.05), identifying a clinically actionable threshold (HSI=35.31) where risk escalation plateaus. Utilizing routinely measured parameters (age, sex, liver function, BMI) (43), HSI emerges as a cost-effective screening tool for population-level diabetes prevention strategies (44). Complementing prior research focused on high-risk management (42) our findings-supported by large-scale validation, simplified risk quantification, and direct primary prevention applications-provide critical insights for early-stage intervention in public health frameworks.

Our study has notable limitations requiring consideration. First, while incorporating representative samples from 11 cities, we could not fully account for regional heterogeneity in dietary patterns (45) and socioeconomic status (46) - factors potentially influencing IFG progression through microbiota-mediated metabolic regulation (47) and psychosocial pathways (48). Second, although excluding baseline diabetes and incident cases, undocumented glucose-altering medications initiated during the mean 3.02-year follow-up period may confound natural dysglycemia progression (49). Third, the retrospective design’s reliance solely on fasting glucose measurements excluded individuals with isolated impaired glucose tolerance, while the absence of oral glucose tolerance tests (OGTT) and hemoglobin A1c (HbA1c) data constrained comprehensive metabolic evaluation. Finally, similar to most single-cohort studies (50), the generalizability of our identified HSI threshold (35.31) requires validation across diverse populations to establish population-specific risk stratification criteria. Future investigations should integrate regional electronic health records for medication tracking, incorporate multi-omics metabolic assessments, and conduct multi-cohort analyses with social determinant evaluations to advance precision prevention strategies.

## 5 Conclusions

This study first reveals a nonlinear HSI-IFG association (plateau risk at HSI=35.31) in Chinese adults. Hepatic markers show early predictive value, particularly for younger populations, suggesting HSI’s utility as a cost-effective screening tool for targeted diabetes prevention in resource-limited settings.

## Data Availability

The datasets presented in this study can be found in online repositories. The names of the repository/repositories and accession number(s) can be found below: https://datadryad.org/stash/dataset/doi:10.5061/dryad.ft8750v.
