# Supplementary figures and images for: Association between hepatic steatosis index and impaired fasting glucose: a multicenter retrospective cohort study in China
Source: Front Endocrinol (Lausanne). 2025 Jun 9;16:1556169. doi: 10.3389/fendo.2025.1556169 (PMC12183071; doi:10.3389/fendo.2025.1556169)

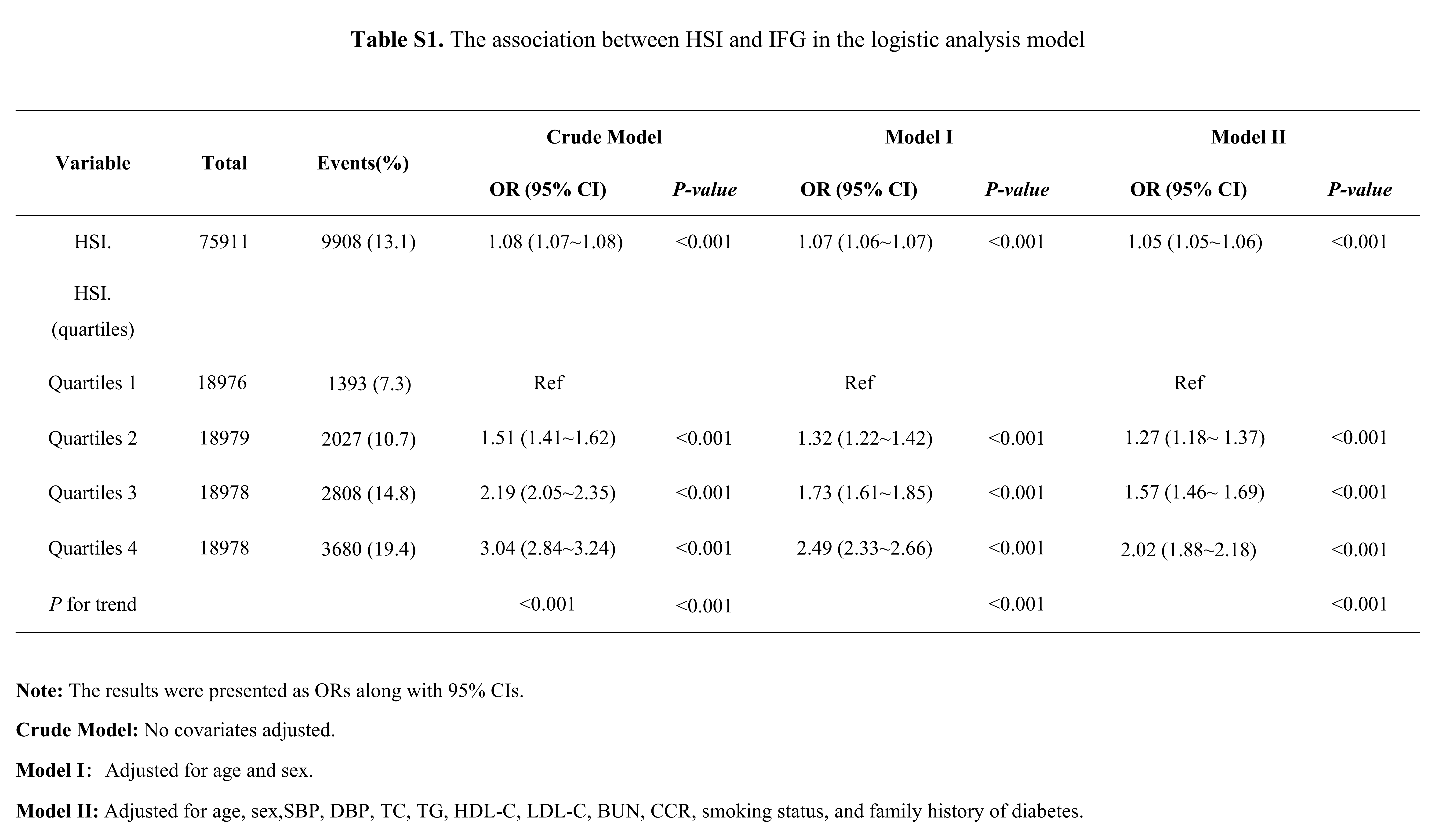

Supplement: Supplementary file 1 [file SupplementaryFile1.tif]
